# Understanding factors influencing uptake and sustainable use of the PINCER intervention at scale: A qualitative evaluation using Normalisation Process Theory

**DOI:** 10.1371/journal.pone.0274560

**Published:** 2022-09-19

**Authors:** Libby Laing, Nde-eshimuni Salema, Mark Jeffries, Azwa Shamsuddin, Aziz Sheikh, Antony Chuter, Justin Waring, Anthony Avery, Richard N. Keers

**Affiliations:** 1 Lifespan and Population Health, School of Medicine, University of Nottingham, Nottingham, United Kingdom; 2 Division of Pharmacy and Optometry, Centre for Pharmacoepidemiology and Drug Safety, School of Health Sciences, University of Manchester, Manchester, United Kingdom; 3 NIHR Greater Manchester Primary Care Patient Safety Translational Research Centre, Manchester Academic Health Sciences Centre [MAHSC], University of Manchester, Manchester, United Kingdom; 4 Faculty of Health Sciences, University of Hull, Hull, United Kingdom; 5 Usher Institute, Edinburgh Medical School, University of Edinburgh, Edinburgh, United Kingdom; 6 Haywards Heath, West Sussex, United Kingdom; 7 School of Social Policy, Health Services Management Centre, University of Birmingham, Birmingham, United Kingdom; Flinders University, AUSTRALIA

## Abstract

**Introduction:**

Medication errors are an important cause of morbidity and mortality. The pharmacist-led IT-based intervention to reduce clinically important medication errors (PINCER) has demonstrated improvements in primary care medication safety, and whilst now the subject of national roll-out its optimal and sustainable use across health contexts has not been fully explored. As part of a qualitative evaluation we aimed to identify factors influencing successful adoption, embedding and sustainable use of PINCER across primary care settings in England, UK.

**Methods:**

Semi-structured face-to-face or telephone interviews, including follow-up interviews and an online survey were conducted with professionals knowledgeable of PINCER. Interview recruitment targeted four early adopter regions; the survey was distributed nationally. Initial data analysis was inductive, followed by analysis using a coding framework. A deductive matrix approach was taken to map the framework to the Normalisation Process Theory (NPT). Themes were then identified.

**Results:**

Fifty participants were interviewed, 18 participated in a follow-up interview. Eighty-one general practices and three Clinical Commissioning Groups completed the survey. Four themes were identified and interpreted within the relevant NPT construct: Awareness & Perceptions (Coherence), Receptivity to PINCER (Cognitive Participation), Engagement [Collective Action] and Reflections & Adaptations (Reflexive Monitoring). Variability was identified in how PINCER awareness was raised and how staff worked to operationalise the intervention. Facilitators for use included stakeholder investment, favourable evidence, inclusion in policy, incentives, fit with individual and organisational goals and positive experiences. Barriers included lack of understanding, capacity concerns, operational difficulties and the impact of COVID-19. System changes such as adding alerts on clinical systems were indicative of embedding and continued use.

**Conclusions:**

The NPT helped understand motives behind engagement and the barriers and facilitators towards sustainable use. Optimising troubleshooting support and encouraging establishments to adopt an inclusive approach to intervention adoption and utilisation could help accelerate uptake and help establish ongoing sustainable use.

## Introduction

Medication errors are an important cause of patient morbidity and mortality [1], with approximately 237 million medication errors being made annually across the National Health Service (NHS) in England, UK [2]. Around 66 million of these are potentially clinically significant (i.e. could result in harm), 34% of which can be attributed to prescribing in primary care (general practice) [2]. Furthermore, approximately 4% of hospital admissions are linked to drug-related morbidity [3, 4] with avoidable drug reactions resulting in 700 deaths/year and contributing to a further 1,700 deaths/year [5] whilst costing the NHS £98.5 million annually [5, 6].

The use of information technology (IT) systems designed for health-care safety purposes can effectively reduce rates of medication errors and associated adverse outcomes [7–9]. Developed with a particular focus on the primary care setting, one evidence-based prescribing safety and medication monitoring intervention is the pharmacist-led IT-based intervention to reduce clinically important medication errors (PINCER).

PINCER, which is intended to be run every six months, consists of the following components [10, 11]:

- A pharmacist trained to deliver the intervention conducts searches on a general practice electronic clinical record system using a set of 13 pre-identified, evidence-based prescribing safety indicators (described in Table 1).
- Using the principles of educational outreach [12], the pharmacist then communicates the search results to general practitioners (GPs) and their teams and helps to devise a collaborative action plan aimed to reduce future medication risk.

**Table 1:** Descriptions of the PINCER intervention indicators [13]*.

Query	Indicator description
Related clinical outcome: gastrointestinal (GI) bleed	
A2	Prescription of an oral non-steroidal anti-inflammatory drug (NSAID), without co-prescription of an ulcer healing drug, to a patient aged ≥65 years
B2	Prescription of an oral NSAID, without co-prescription of an ulcer healing drug, to a patient with a history of peptic ulceration
B3	Prescription of an antiplatelet drug without co-prescription of an ulcer-healing drug, to a patient with a history of peptic ulceration
C2	Prescription of warfarin or direct oral anticoagulant (DOAC) in combination with an oral NSAID
D2	Prescription of warfarin or DOAC and an antiplatelet drug in combination without co-prescription of an ulcer-healing drug
E2	Prescription of aspirin in combination with another antiplatelet drug (without co-prescription of an ulcer-healing drug)
Related clinical outcome: heart failure	
F2	Prescription of an oral NSAID to a patient with heart failure
Related clinical outcome: acute kidney injury	
G2	Prescription of an oral NSAID to a patient with an estimated glomerular filtration rate (eGFR) <45
Related clinical outcome: exacerbation of asthma	
H2	Prescription of a non-selective beta-blocker to a patient with asthma
Monitoring indicators	
I2	Patients aged 75 years and older who have been prescribed an angiotensin converting enzyme (ACE) inhibitor or a loop diuretic long term who have not had a computer-recorded check of their renal function and electrolytes in the previous 15 months
	Patients receiving methotrexate for at least 3 months who have not had a recorded:
J2	• Full blood count (FBC) within the previous 3 months (J2)
J3	• Liver function test [LFT] within the previous 3 months [J3]
K2	Patients receiving lithium for at least 3 months who have not had a recorded check of their lithium concentrations in the previous 3 months
L2	Patients receiving amiodarone for at least 6 months who have not had a thyroid function test (TFT) within the previous 6 months

*Information obtained from pages 22–23 of the PINCER National Rollout, Progress Report to NHS England and the AHSN Network, July 2020 [13]

PINCER has thus far been tested and shown to be effective in a cluster randomised controlled trial conducted between 2006–2010 [10]; was successfully implemented on a larger regional scale between 2013–2017 [13] and, as of 2018, is currently being rolled out nationally across the English NHS.

Previous process evaluations of PINCER, undertaken during the trial phase have focused on stakeholders’ views on acceptability, the potential impact and strategies for optimising wide scale roll-out [14], cost-effectiveness [15] and pharmacists’ assessment and time implications of undertaking intervention specific training [16]. This covered the three counties of England where trial participants were recruited from [10]. Intervention effectiveness, acceptability and feasibility of implementing PINCER in different settings as well as contextual factors that could impact on its use and effects were also assessed from the scaling up phase which was conducted across one region of England in which one of the counties from the trial is located [13]. More recent work aimed to identify overarching strategies that could support implementation and sustainable use of prescribing safety indicator based interventions, which although included PINCER was not intervention specific [17]. Informed by the Normalisation Process Theory (NPT) [18], the study reported in this paper investigated longitudinally how PINCER was understood and operationalised during the national roll-out phase with the aim of expanding on the aforementioned evaluations and identify underpinning factors that could help facilitate the success of implementation and sustainable use across different primary care settings on a national level.

## Methods

### Theoretical framework: Normalisation Process Theory

NPT is a middle range theory which focuses on the work people do and the social practices and processes involved when implementing, embedding and integrating a new intervention into the environment where it is intended to be used [18–20]. It offers a framework which can be used to gain an understanding of how individuals and groups work to make sense of an intervention, the new practices involved and how people collaborate to operationalise and sustain its use in practice [18, 20–22]. NPT consists of four constructs, Coherence (meaning and sense-making), Cognitive Participation (commitment and engagement), Collective Action (making the intervention function) and Reflexive Monitoring (reflecting and appraising the intervention) [18, 20–22], the main components of which are outlined in Table 2.

**Table 2:** Components of the four Normalisation Process Theory constructs [18, 20–22].

**Coherence—**meaning and sense-making by participants
How participants define and make sense of an intervention
Gaining an understanding of how it differs from other interventions
Developing a shared understanding of the intervention and how to integrate it into their workplace
Understanding what tasks are involved on an individual and group level
Seeing the value, importance and benefits of it
**Cognitive Participation—**commitment and engagement by participants
How key participants work to drive a new set of practices forward
How people rethink and reorganise themselves as a group to contribute to the required new ways of working
Belief in the intervention and the validity of their contribution to it
Defining the actions and procedures required for the sustainability of the intervention in practice
**Collective Action—**the work done by participants to make the intervention function
The interactional work participants do with each other when operationalising the intervention in practice
Taking responsibility and building confidence in the intervention and their own and others’ involvement with it
Division of labour including allocation of tasks to those with the required skill set
Managing the new set of practices in alignment with policies and protocols and with allocated or existing resources
**Reflexive Monitoring–**participants reflect on or appraise the intervention
Determining the effectiveness and usefulness for themselves and others
Evaluating the worth of the intervention
Appraisal of the impact on their own work and within the context that it has been operationalised
Attempts made to redefine or modify the intervention and/or how it is used

The constructs work in a non-linear, dynamic manner with one another and within the broader contexts including social norms, group culture, policies and protocols and the organisational structures in which the intervention is being actioned [18].

The NPT has been used successfully in previous prescribing safety evaluation studies [23, 24], including a complex, pharmacist-led intervention that aimed to identify patients at risk from potentially hazardous prescribing through the use of an electronic audit and feedback surveillance dashboard [25]. In this current study the NPT was considered the most appropriate framework to help identify and understand factors that can lead to the successful implementation, embedding and continued sustainable use of the PINCER intervention.

### Study design

This study, which was part of a larger project aiming to evaluate the clinical effectiveness and cost effectiveness of PINCER, was a medium-long term qualitative process evaluation of the use of PINCER during the national roll-out phase. It was conducted using semi-structured, individual and group interviews and an online national survey. A mixed-methods approach was taken in order to capture in-depth data from regions that had been using PINCER in the medium-longer term and collate these with other widespread and diverse views from regions where PINCER had been implemented at varying time points during the roll-out.

#### Interviews

*Recruitment of participants*. Recruitment targeted four Academic Health Science Network (AHSN) regions of England, UK where there had been early adoption of PINCER. The sampling frame included stakeholders who, for the purposes of this work, were intervention developers, personnel responsible for the national roll-out and AHSN staff. Clinical Commissioning Group (CCG]) and general practice staff who had experience in implementing and/or running the intervention either in the medium-term (6–18 months) or long-term (>18 months) timeframe were also invited to participate. Intervention developers and personnel involved in the roll-out of PINCER were approached directly by NS or LL. Contact was made with the AHSNs, by LL or NS, via the relevant Research and Development (R&D) department if they were governed by an NHS trust or their business manager if they were a Company Limited by Guarantee. Details of the study and expression of interest (EOI) forms were circulated to CCG and general practice staff by the relevant Clinical Research Network (CRN). Those who responded to the EOI were then contacted by LL or NS. All participants who agreed to take part were provided with an information sheet and had the opportunity to ask questions prior to providing written informed consent. A £20 high street voucher was offered per interview as a reimbursement for the participant’s time. At the end of the initial interview, participants were asked if they would like to take part in a follow up interview, which were scheduled approximately 6–12 months after the initial interview.

#### Data collection

Semi-structured interview templates (provided in S1 Appendix), informed by the NPT and experience of previous evaluations conducted by the team were used to guide the interviews. Initial interviews were conducted between June 2018 and November 2020 and the follow-up interviews between November 2019 and February 2021. The interviews were audio-recorded and took place either face-to-face in the participants’ place of work or over the telephone depending on practicalities and in line with COVID-19 social distancing guidelines at the time of interview. The interviews were conducted by LL, a Research Fellow with a previous nursing and research background or NS, a Senior Research Fellow and pharmacist both of whom have experience in qualitative health-care research. Fifty participants from 27 organisations across the four English AHSN regions were interviewed including: two intervention developers, three personnel involved in the PINCER roll-out from one organisation, five AHSN employees from four AHSNs, six CCG employees from five CCGs and 34 general practice staff from 18 general practices. Their engagement with the intervention had been long-term for 16 organisations, medium-term for 10 establishments and short-term for one who disclosed they were no longer using PINCER at the time of interview. Follow-up interviews were conducted with 18 participants from 12 organisations including four AHSN employees from three AHSNs, five CCG employees from four CCGs, and eight general practice staff from four general practices. Details of organisation type, participant job roles and if they participated in a follow-up interview are given in Table 3 (more specific details, including involvement with PINCER can be found in S1 Table).

**Table 3:** Interviewee details.

Organisation type	Job Role*	n =	Participated in follow-up
AHSN*	Area 1	1	Y
	Area 2	1	Y
	Area 2	1	Y
	Area 3	1	N
	Area 4	1	Y
CCG	Senior Innovation Project Lead /CCG Pharmacist	1	Y
	Chief Pharmacist	1	Y
	Locality Lead Pharmacist	1	Y
	Medicines Optimisation Technician	1	Y
	CCG Prescribing Support Pharmacist	1	N
	Pharmacy Technician	1	Y
General Practice	GP	9	Y (n = 2)
	Practice Manager	6	N
	Clinical Pharmacist	6	Y (n = 3)
	Practice Pharmacist	3	Y (n = 2)
	Primary Care Network (PCN])Pharmacist	2	N
	Medicines Optimisation Pharmacist	1	N
	Lead Dispensers	2	N
	Medicines Manager	1	N
	Data Lead	1	Y
	Advanced Nurse Practitioner	1	N
	Practice Manager/Practice Nurse	1	N
	Medical Secretary	1	N

*Job titles of AHSN staff have been withheld due to the possibility of participant identification

LL conducted initial interviews with 40 participants and follow-ups with 16, NS conducted initial interviews with 10 participants and follow-ups with two. The initial interviews duration ranged between 10–69 minutes (mean = 37 minutes), the follow-up interviews duration ranged between 18–40 minutes (mean = 27 minutes).

#### National survey

*Recruitment of participants*. Recruitment targeted all regions of England, UK. All 15 CRNs in England were approached for assistance, 11 of whom were able to offer support. Study details were circulated with EOI forms by the 11 CRNs to the CCGs and practices within their region. Personnel responsible for the roll-out also disseminated study details to relevant contacts. Primary care employees who identified themselves as having any experience of the PINCER intervention were invited to complete the survey. To complement and augment the interview data, staff who had experience of the intervention in the short-term (<6 months) were also included in the survey. Those who agreed to take part were provided with an information sheet and given the opportunity to ask questions prior to being sent a link to the survey. In order to capture a broad range of experiences and opinions, respondents were encouraged to complete the survey in collaboration with colleagues where possible. Completion was deemed as implied consent. Due to the brevity of the survey, reimbursement was not offered.

#### Data collection

Survey questions reflected those asked in the interviews, presented in questionnaire form (provided in S2 Appendix). The survey was run on Jisc online surveys software (https://www.onlinesurveys.ac.uk/) from October 2020 until April 2021. Unique links to the survey were generated and emailed to respondents via Jisc software. Non-responders received up to three reminders to complete the survey.

From the 99 individuals who agreed to participate on behalf of their organisation and in collaboration with colleagues where possible, a total of 84 surveys were completed on behalf of 81 practices by 98 respondents and three CCGs by three respondents. Forty-three of the 84 responses came from establishments within the four AHSN regions covered by interview recruitment (Area 1 n = 28, Area 2 n = 7, Area 3 n = 5 and Area 4 n = 3). At the time of survey completion, 60 of the 84 establishments were currently using PINCER. Of the 24 that were not currently using it, 15 had used it previously. Of the practices who were currently using it or had used it previously, 33 (29 current and 4 previous) had used it in the long-term, 29 (23 current and 6 previous) had used it in the medium-term and 12 (eight current and four previous users) had used it in the short-term timeframe. One practice who were previous users did not disclose the duration of their involvement with PINCER. Two of the three CCGs were currently using PINCER and had been using it in the long-term, the other had used it previously within the short-term timeframe. General details of organisation type and job role of respondents are given in Table 4 (more specific details, including which anonymised AHSN region they were recruited from and time involved with PINCER, can be found in S2 Table).

**Table 4:** Survey respondent details.

Organisation type	Job Role	n =
CCG	CCG Pharmacist	2
	CCG Pharmacy Technician	1
General Practice	PCN Pharmacist	25
	GP	24
	Practice Pharmacist	19
	Practice Managers	18
	CCG Pharmacists	5
	Practice Nurses	3
	Senior Manager	1
	Clinical Pharmacist	1
	PCN Pharmacy Technician	1

#### Data analysis

An inductive approach was taken initially which was guided by the six phases of qualitative analysis described by Braun and Clark (2006) [26]. All interview audio-recordings were re-played and crosschecked with the interview transcripts in order to correct errors and to re-familiarise NS and LL with the data set at the start of the analysis. QSR NVivo 12 Pro was used to support the organisation of the data. Initial coding of the interview transcripts was conducted by NS and second coding was done by LL independently. Following discussion between NS and LL a coding framework was developed and agreed on with members of the wider team (RK, MJ & AC). The framework was then used to code the follow-up interview transcripts and survey data, with adjustments and code refinement being made during this phase. Following further discussions between LL and RK on the amended framework and completed coding, a more deductive matrix approach was then used to map the coding framework to the four constructs of the NPT. Results of the mapping were then discussed between LL, RK and MJ following which themes were interpreted across the full data set.

### Ethical approval

Ethical approval was granted by the East Midlands–Nottingham 2 Research Ethics Committee and the NHS Health Research Authority (IRAS ID: 212446).

## Results

### Themes

Four themes were identified, Awareness & Perceptions, Receptivity to PINCER, Engagement and Reflections & Adaptations. Although they were interrelated, each theme mapped predominantly to one construct of the NPT (as shown in Table 5).

**Table 5:** Alignment of themes with the Normalisation Process Theory constructs.

**Coherence—Awareness & perceptions**	
Becoming aware	There were some indications of curiosity in PINCER and to find out more based on some initial and informal introductions to the concept of the intervention e.g. at conferences.
	Social media, in particular Twitter, facilitated communication on the use of PINCER and highlight the benefits amongst pharmacists. This helped escalate enthusiasm to be trained and engage with the intervention.
	For others, the process was less pro-active and the information they received on PINCER came from top down, for example, when it had been decided to include it in the organisation’s medicines optimisation programme.
Understanding of the PINCER intervention	Being included in the Quality and Outcomes Framework (QOF) 2019/20 was influential in driving PINCER forward, encouraging uptake and helping to clear up misunderstandings on what PINCER is, including the use of ‘PINCER alternatives’.
Inclusivity & collaboration	There were signs of staff working together and sharing tasks relating to PINCER with some taking a full practice approach whereas others only included or informed certain staff members on its use.
Perceived benefits & drawbacks	Cost implications were seen as both a positive and negative depending on understanding surrounding it. Some thought it would help de-prescribe and thereby be cost-effective whereas others expressed concerns around cost of use.
	Being pharmacist-led was seen by some to be beneficial for general practitioners’ (GPs) workload however there was also concern expressed about handing over some ownership of care from the GPs to the pharmacists.
**Cognitive Participation–Receptivity to PINCER**	
Stakeholder interaction	Motivated personnel and good collaborations between intervention developers, personnel responsible for the roll-out and the Academic Health Science Networks (AHSNs) helped to boost enthusiasm and uptake of PINCER.
	Top down, structured and tailored communication which filtered from the AHSNs through the Clinical Commissioning Groups (CCGs) to practice level had a key role in raising awareness and facilitating buy-in.
Influence of evidence	At the start of the roll-out, the publication of the PINCER trial in the Lancet was seen to have helped ‘sell’ PINCER as did discussions surrounding the evidence-base during the training sessions, particularly around the indicators.
	The evidence on the uptake and success of PINCER as the roll-out progressed was considered powerful and helpful in mitigating arguments against its use.
Incentives & inclusion in policy	Offering/receiving incentives to use PINCER helped promote its use and increase uptake.
	No longer having a directive reason for its use discouraged continued use for some, e.g. once it had helped fulfil the requirements of QOF for those who had only adopted it for that purpose, it was no longer considered relevant or a priority.
Capacity & Contextual factors influencing decisions to adopt and use PINCER	There was some apprehension with some feeling threatened by using PINCER in relation to workload demands, perceived resources required and capability. This impacted on uptake with some resisting adoption and others working through how to implement it and make it work within their organisation.
Fit with own & organisational objectives & values	Some perceived PINCER as being unnecessary or an additional activity that would need to be incorporated into their existing workload whereas others viewed the principles of it and its use as being an integral and fundamental part of their role.
	PINCER was seen by many as a useful tool that could help meet the organisational objectives of prescribing safely and protecting patients from harm.
**Collective Action–Engagement**	
Training	The type of training undertaken varied from being official PINCER training, to in-house training or no training. Training type and level of engagement with training activities did not seem to correlate with the extent and duration of use.
	There were indications of some feeling confident in their abilities and/or persevered to become self-taught.
	There were reports of pharmacists continuing to actively seek training after the declaration of the COVID-19 pandemic.
Implementation & running PINCER	Accessibility of troubleshooting support in the implementation stage and for any subsequent issues encountered varied across establishments. Some felt that there was good support offered by stakeholders, CCGs as well as between peers, whereas others found it more difficult to access any support either through formal or informal routes.
	Problem solving was evident in which staff would work together to overcome issues and make PINCER processes work in practice. This included helping one another with technical issues, giving praise for overcoming difficulties and also allowing some ring-fenced time for the staff member responsible to run the searches.
	There were differences in how many components of PINCER were reportedly used, participants often made selections based on practice demographics and priorities.
	Although there was still some intention to engage with the intervention during the pandemic, remote working made this more difficult.
Organisational structure & timing	Merging of CCGs had implications for continuing with and streamlining the use of PINCER.
	Formation of Primary Care Networks [PCNs] increased the amount of pharmacists available to run PINCER however, due to timing with COVID-19 and ASHN funding for licence fees and training costs coming to an end, training was harder for the trainers to deliver and less accessible to potential participants.
	In some regions, communication between CCG and PCN pharmacists was reduced with CCG staff being no longer aware if the PCN pharmacists are engaging in PINCER work.
	However, PCN pharmacists also acknowledged how PINCER fitted with their wider agenda with some choosing to actively use it to assist with Structured Medication Review (SMR) work.
Signs of embedding & commitment	System changes had been implemented which included putting alerts on the clinical system, adding information to medicine labels, instilling and sustaining new processes for organising monitoring indicator related blood tests. There were also reported changes to communication with patients and thought processes when prescribing.
	Continuing to use PINCER throughout unpredicted and more challenging times was indicative of commitment of use.
**Reflexive Monitoring–Reflections & adaptations**	
Reflections on use	Experiences of ease or difficulty of use and usefulness/effectiveness impacted on motivation for continued engagement.
	Information technology (IT) issues were still evident which, although some had tried to resolve, prevented PINCER from being utilised fully by others.
Suggested adaptations	There were suggestions/recommendations of adding indicators or changing the ones available to make it ‘more current’.
	There were also some intentions to use PINCER as an educational tool for less experienced prescribers.

Where relevant and to provide more context, fuller (reference) quotes that excerpts in the text below have been taken from are provided in S3 Table.

#### Awareness & perceptions: Coherence

This theme mainly relates to how participants became aware of PINCER, how they developed an understanding of it and how able they were to differentiate it from other prescribing safety interventions. It also covers how participants integrated PINCER into their workplace and what their perceived or experienced benefits or drawbacks of its use were.

*Becoming aware*. Participants reported varying levels of effort made in order to gain an awareness of PINCER and share this knowledge and potential enthusiasm for the intervention amongst colleagues and peers. For some, awareness was raised through more top-down, directive methods including being informed of it as it was being included in their organisation’s medicines optimisation programme. For example, a questionnaire response stated that they became aware of and adopted PINCER … *As [it was] recommended by the CCG to monitor prescribing safety …* **Practice O, Area 6 (Respondent–GP)**

Interest and curiosity generated from informal introductions led to others seeking out more information about it with social media interaction facilitating positive peer influence.

“*… the pharmacy world is actually quite engaged with Twitter … and having people post messages about you know how easy they have found it to use*, *how straightforward it was*, *how they felt they were protecting patients*, *those kinds of things actually did help [to drive the intervention forward]…*” **AHSN employee, Area 2**

*Understanding of the PINCER intervention*. Understanding of the intervention varied amongst participants both between and within organisations. At the beginning of the roll-out, misunderstandings surrounding PINCER led to some staff thinking they were using PINCER when they were not and others knowingly using non-PINCER prescribing safety review techniques which they perceived as being valid alternatives to PINCER. Effort was made by stakeholders, in particular AHSN staff, to allay these misunderstandings, address reluctance to change from what was being used if it was not PINCER and ensure the intervention was driven forward as intended.

“*… there was a lot of talk of well I am doing PINCER or I am doing the equivalent of and that wasn’t defined anywhere*, *nobody had spelt that out so we had to do a lot of work on saying well this is what PINCER is and this is what PINCER isn’t so an awful lot of misunderstanding if I am honest that we have had to spend hours and hours sorting out*.” **AHSN employee, Area 2**

In support of the World Health Organisation’s (WHO’s) 2017 Medication Without Harm campaign [27], the Department of Health and Social Care, England formed a Short Life Working Group which advocated the use of PINCER in primary care [28]. Its use was then subsequently incorporated into the Quality and Outcomes Framework (QOF])2019/20 [29], which helped mitigate misunderstandings on PINCER and the use of PINCER alternatives as well raise its profile amongst primary care staff.

*“… and obviously it going in to QOF and … the Patient Safety Strategy*, *those things were enormously helpful … I think the other thing that was really helpful in policy terms was the World Health Organization Global Challenge …*” **AHSN employee, Area 2 (Quote 1, S3 Table)**

The ability to differentiate PINCER from other interventions also varied; although most seemed to have a good awareness of what the PINCER indicators were, when participants discussed or listed PINCER activities a few also mentioned engaging in activities aimed to reduce anticholinergic burden and opioid use which are not part of the PINCER intervention.

*Inclusivity and collaboration*. Some participants reported that their place of work had adopted an inclusive approach in which there was an open awareness of the use of PINCER and relevant intervention tasks had been shared amongst different team members. However, although less commonly reported, there were other organisations in which information of its use, including the reason for adopting and the responsibility of running it had been shared amongst certain staff members only. For instance, one survey response from a practice which had been using PINCER medium-term stated that there was a … *“Discussion of individual indicators at Monthly Clinical Governance Meetings … [attended by] All practice staff within the practice”* **Practice B, Area 5 (Respondents–Practice Pharmacist & PCN Pharmacist).** In contrast another respondent, whose practice had been using it in the short-term, reported that their practice was currently using PINCER but they were *“not sure why”* **Practice U, Area 6 (Respondent–Practice Nurse)**

*Perceived benefits and drawbacks of PINCER*. Participants held different perceptions surrounding financial implications. Some considered PINCER to be a de-prescribing tool which could help reduce prescribing associated costs whereas others expressed concerns around the potential costs of adopting and running PINCER such as licence fees. These perceptions tended to be given in combination with other reasons or justifications as to why they were currently using PINCER or not.

“*We have struggled with doing this at the beginning of the COVID pandemic and we were under the impression that we would be charged to access the software …*” **Practice A, Area 4 (Respondent–Practice Pharmacist)**

Being pharmacist-led was viewed positively by many in the respect that it could reduce GP workload; however, there was some apprehension about handing over elements of care from GPs to pharmacists.

*“… we have to be part of a big team with a pharmacist on board and we have got to you know … hand over some of the responsibility and some of the ownership of these patients and you know*, *for some doctors it is fine … but some doctors*, *they don’t find it easy …*” **GP, Practice 2, Area 3**

#### Receptivity to PINCER: Cognitive participation

This theme covers how stakeholders (i.e. intervention developers, personnel responsible for the roll-out and AHSN staff) interacted with one another other and personnel who would be implementing and using PINCER. It also covers how influential the available evidence on the effectiveness of PINCER was as well as the advantages of being able to offer or receive incentives for its use and the inclusion of PINCER in policy. Reasons for choosing to use and continue to use PINCER are also given.

*Stakeholder interaction*. Good collaborations in relation to ensuring the success of the roll-out, motivated personnel and cohesiveness amongst stakeholders also helped to raise interest and enthusiasm in adopting PINCER both on a stakeholder level and from the top down.

*“… [X] AHSN and senior staff members of [Y] AHSN [have] just done a phenomenal job with working with us [intervention developers] and [personnel responsible for the roll-out] to ensure that roll-out is going successfully and it has now just got this fantastic head of steam*, *there is a lot of enthusiasm around for it*, *the AHSNs are on board*, *even ones that … had problems or reservations early on seem to be really positive about it*…” **Intervention developer, Area 1**

This included having a shared belief in the intervention, putting in extra effort and working across establishments to overcome any difficulties encountered. As one participant explained … “*We worked really*, *really hard to keep the show on the road*.*”* **AHSN employee, Area 2 (Quote 2, S3 Table)**

Strategic, tailored communication that filtered down from ASHNs to CCGs through to practices also had a key role in facilitating buy-in.

*“The CCG’s … [were] sort of drip feeding evidence*, *information*, *… obviously with all of it we try and sort of include the key bits of evidence and references and so forth but yes it has been mostly sort you know cascaded information …*” **AHSN employee, Area 3 (Quote 3, S3 Table)**

*Influence of evidence*. At the start of the roll-out, the publication of the PINCER trial in The Lancet [10] and discussions around the evidence-base of the indicators during the training helped enhance credibility and acceptability amongst staff.

*“… I think the publication in The Lancet was really important because it got back to a sort of wider platform and it just had that sort of rigour about it*.” **AHSN employee, Area 2**

As the roll-out progressed, the available evidence on the uptake and success of PINCER was considered important in mitigating arguments against its use and driving it forward.

*“… so I think the biggest thing that has happened in the last 12 months*, *obviously COVID aside*, *is that we got the beginnings of the data from the interim report … That has been a game changer … we know what happened before*, *we know what happened afterwards and that has been hugely*, *hugely powerful*.” **AHSN, employee, Area 2, Follow-up interview (Quote 4, S3 Table)**

*Incentives and inclusion in policy*. Being able to offer incentives helped stakeholders encourage uptake as well as increase motivation amongst those receiving the incentives to engage with the intervention. This had a noticeable interrelationship with policy, for example, QOF advocating the use of PINCER and other prescribing schemes that offered financial rewards.

The following quote gives an example of financial incentives influencing decisions at practice level in regards to adoption and implementation of CCG driven initiatives such as PINCER … *“You don’t have to [go with what the CCG drive forward] but if you want their payments …*” **Practice Manager, Practice 3, Area 3 (Quote 5, S3 Table)**

No longer having policy and/or incentive driven reasons to use PINCER discouraged further use for some, for example, a practice stated that they had … “*Used [PINCER] previously for prescribing QOF indicators so [it was] no longer relevant*” **Practice B, Area 1 (Respondent–Practice Manager)**

*Capacity and contextual factors influencing decisions to adopt and use PINCER*. In terms of workforce capacity and context, a notable amount of apprehension was shown surrounding additional workload demands, resources required, and the capability needed to be able to operationalise PINCER. Amongst those who gave capacity and contextual factors as being a barrier towards its use, as well as there being awareness of the benefits, there were also still some indications of intentions to engage with the intervention to some extent.

*“I think right now if that [continuation of use of PINCER] was presented to us*, *it would be difficult to take on board because we’re having to use another tool*, *we’re enforced to use that tool … but if it highlighted significant improvement or you know and it was very easy to use then we maybe would consider that*…*”* **Clinical Pharmacist 2, Practice 1, Area 1**

A survey respondent listed mainly capacity or contextual related reasons for not currently using PINCER which included service delivery issues during the pandemic.

*“Large practice size of over 20*,*000 patients and 10 GPs*, *only one clinical pharmacist at present*. *Waiting for the Pharmacy Team to expand before utilising PINCER*. *Other medication safety programmes such as Eclipse are in … PINCER was also not considered a priority as lack of access to phlebotomy services during the COVID-19 pandemic*…” **Practice D, Area 3 (Respondent–Clinical Pharmacist) (Quote 6, S3 Table)**

The above quote also highlights perceptions and understanding around competing systems in which there was some thinking that if you use one type of medication safety intervention, you do not have to use another.

*“We’re not using it [PINCER] anymore within [name of] CCG*, *they use a tool called Eclipse which I believe uses the same parameters as PINCER but just pulls them out into a different program*.” **Clinical Pharmacist 1, Practice 1, Area 1**

*‘Fit’ with own and organisational objectives and values*. A few participants viewed PINCER as being an extra activity that would need to be incorporated into their existing workload whereas others, mainly pharmacists or pharmacy technicians, viewed the intervention and principles behind it to be a fundamental and integral part of their role.

*“*… *It just means extra work … given a list of patients that you know someone has landed on my lap*, *if I don’t deal with them*, *they come to harm then they say well it was on your lap …*” **GP, Practice 2, Area 3 (Quote 7, S3 Table)**

There was also evidence of shared buy-in and decision making to continue to use PINCER amongst some who initially had a more directive reason for adopting it but who experienced the benefits and acknowledged that it fitted with a wider agenda relevant to their roles.

“[reason given for adoption] … *Originally part of [a] CCG prescribing initiative scheme* … *PCN [Primary Care Network] pharmacists have continued to run these searches for patients quarterly to ensure safety indicators [are] reviewed …”* They also stated that “… *they [the searches] are very beneficial to practices especially as it links into the PCN DES [PCN Network Contract Directed Enhanced Service] for high risk medication and safety principles and the PCN pharmacists agreed it would be a good work stream to continue with* …*”* **Practice J, Area 6 (respondents—PCN Pharmacist & Practice Manager)**

#### Engagement: Collective action

This theme covers how participants interacted with PINCER and illustrates factors that participants felt made using PINCER easier or more difficult. It also highlights signs that the intervention was being embedded into everyday practice.

*Training*. Official PINCER training was offered and facilitated by personnel responsible for the roll-out. Some participants actively sought to access this training whereas others were offered the opportunity to participate either by the AHSN, CCG or colleagues. Accessing the training in a more active or passive manner often related to the reason for adopting PINCER.

*“… so PINCER has come in at CCG level and … then so that is why in April … we got ‘right this is the new PQS [Pharmacy Quality Scheme] for the year’ and part of that was [the lead dispenser] … went away and did … some PINCER training …*” **Practice Manager, Practice 3, Area 3**

There did not appear to be a relationship between training type undertaken, level of engagement with training activities and the extent and duration of intervention use. Some participants appeared confident in their abilities and/or persevered to become self-taught.

Similarly, there were also some reports of pharmacists actively seeking training amidst the pandemic and from areas where PINCER was not supported by the CCG.

*Implementing and running PINCER*. Accessibility of troubleshooting support for those who required it, either at the implementation stage or during subsequent use, varied across organisations. There were some participant accounts of satisfactory support being offered by stakeholders and CCG staff as well as between peers whereas others found it more difficult to access adequate support either through formal or informal routes. Difficulty in accessing support, dissatisfaction with support received alongside other workforce pressures led to participants continuing with attempts to resolve issues themselves, or to only use the PINCER components that they were able to operationalise and/or feeling less motivated or able to make the intervention function within their organisation. The following quote gives an example of a CCG employee who had continued to advocate the use of PINCER gastrointestinal (GI) indicators during the pandemic, but had experienced difficulties in trying to run it on an upgraded clinical system. There had been no response to emails requesting official support and the COVID-19 vaccination roll-out then took precedence over attempts to resolve this issue.

*“*… *So*, *I haven’t managed to make it run yet*, *and then obviously with the vaccine*, *things have taken over ever since*…” **CCG Prescribing Support Pharmacist, CCG1, Area 3, FU interview (Quote 8, S3 Table)**

Problem solving activities included staff members working together to overcome technical issues, giving praise to those who managed to overcome difficulties and recognising that protected time to engage in PINCER activities was necessary for those undertaking them. For instance, a GP stated that *“… you do need to actually set aside time to just sit down not interrupted …”* **GP/Prescribing Lead, Practice 4, Area 3 (Quote 9, S3 Table),** whilst appreciating that a substantial amount of PINCER related work is undertaken by the pharmacists.

In addition to experiencing operational difficulties, there were other reasons as to why components of PINCER were used variably, and not always in their entirety, across organisations. Addressing all 13 prescribing safety indicators simultaneously within PINCER was considered resource heavy; participants often selected which indicators they were going to run based on relevance to priorities in the prescribing safety agenda and their patient population. There were indications from some participants that they intended to keep using the same, select group of indicators they had chosen from the 13, whereas others planned to or had been working through all 13 in a systematic manner over time.

*“… at the moment the main one that we focus on … is indicator E2 … that was one that we went into detail on that was actually done as a full in depth root cause analysis*, *it was presented as a PowerPoint presentation at one of our GP meetings*, *and then … the sort of end of it was left open to discussion with all clinical colleagues so with a view to devising an action plan as to how we were going to help these patients essentially and make changes within our system in practice*, *so the idea going forward now is that we are going to do that for each and every one of the indicators …*”*…* **Clinical Pharmacist, Practice 1, Area 3 (Quote 10, S3 Table)**

Although there was some intention to engage in the intervention as normal, remote working due to the pandemic made usual interactions with colleagues more challenging.

*“… I made all of the presentations up but we never presented it just because … we started all working from home … so that aspect where we discussed it with the partners*, *that is the other bit that is still on my agenda to do that … if we didn’t have COVID it would have been a lot easier to do it*.” **Practice Pharmacist, Practice 4, Area 3, FU interview**

*Organisational structure and timing*. A few participants experienced or perceived opportunities and difficulties relating to contextual factors such as re-structuring.

*“… all six of the [Area 1] CCGs have come together*, *they now form as one CCG … not only is there this sort of variation around uptake and engagement with PINCER but a lot of that is also dependant on the capacity of the medicines teams in what were previously each of those six old CCG localities … so going forwards in the single CCG … part of that reorganisation is looking at redistributing the practice pharmacists in a more equitable manner so … there may be some opportunity to look at [a] more consistent approach to PINCER you know … bringing up everybody to a similar standard*…” **Chief Pharmacist, CCG 1, Area 1, FU interview**

The formation of Primary Care Networks (PCNs) was seen to increase the amount of pharmacists available to run PINCER however, due to the timing with the pandemic and AHSN funding for training costs coming to an end, training was harder for trainers to deliver and for staff to access.

*“The challenge we have got is just as the AHSNs are coming to the end of our run with this work*, *we have got this whole army of people [PCN pharmacists]*, *new people who need training and that is a massive challenge*. *We have got kind of six months to get as many of them through the process as possible but of course they are busy and there is COVID and you know you could only train so many people at a time and we’re doing it all online … and can only really have like I guess 15 or so tops*, *so it is going to be a slower process …*” **AHSN employee, Area 2, FU interview**

Efforts made by the CCGs and PCNs to communicate with each other, including offering or accessing support were also variable.

*“Yes so the PCN’s have formed*, *we have had very little to do with PINCER since our last phone call [initial interview] … it has certainly dropped off the radar in a PCN practice perspective from us guys … they may still well be doing it however I wouldn’t be convinced because I know a lot of them had issues with Chart Online originally and we haven’t had any noise from the practices … asking any question so it would make me think that they are probably not continuing … Haven’t continued but obviously with COVID-19 a lot of things are … fallen by the wayside*.” **Pharmacy Technician, CCG1, Area 4, Follow-up interview**

However, a facilitating factor in the formation of PCNs was the requirement to sign up to the Network Contract Directed Enhanced Service (DES) Contract Specification 2020/21, first introduced in July 2019 [30]. One of the new services introduced in the PCN DES was conducting Structured Medication Reviews (SMRs) [30] which some of PCN pharmacists were actively using the PINCER tool for. For example, *“…when I do my structured medication reviews … I have been using PINCER … so I was using PINCER to you know flag up some patients …”* **PCN Pharmacist, Practice 7, Area 3, Follow-up interview.**

*Signs of embedding and commitment*. System changes had been made by some, mainly those who had been using PINCER in the medium-longer term, in an attempt to ensure good prescribing safety procedures were adhered to and/or optimised. For example, as a Data Lead and GP explained *“… we have put alerts on …”* **Data lead & GP, Practice 5, Area 2 (Quote 11, S3 Table),** thereby indicating efforts to embed it into the clinical system as well as to provide prompts for safer prescribing that could be used in the longer term.

Such changes also extended to taking actions towards enhancing administration procedures, for instance … *“A Clinical letter [was] created for medicines reconciliation …* **Practice A, Area 4 (Respondent–Practice Pharmacist) (Quote 12, S3 Table),** in addition to there being reports of embedded changes in communication and thought processes whilst prescribing:

*“… actually it is stuck in the back of my head now so I will automatically go hang on a minute you’re on [a] dual antiplatelet*, *we don’t have a PPI [proton pump inhibitor] or gastro protection*, *let’s make sure you’ve got that …*” **Practice Pharmacist, Practice 2, Area 3**

Although many participants, particularly those who had been using PINCER in the medium-longer term, had to alter or reduce how they used PINCER due to the impact of the pandemic, commitment of use was indicated by continuous engagement with PINCER activities through this challenging and unpredictable time.

*“… we have still been running it [PINCER]*, *we ran it during the first lockdown … we literally focused on the care home patients … We have [also] used it for some of the monitoring to make sure that the patients are still getting their bloods … I think the plan was always to use it and we probably will carry on using it …*”

**Medicines Optimisation Technician, CCG1, Area 2, FU interview**

#### Reflections & adaptations: Reflexive monitoring

This theme reports on participants thoughts on PINCER after they had started using PINCER and presents their suggested adaptations for enhancement of the intervention and its usefulness.

*Reflections on use*. Reflections on use were generally positive with negative aspects mainly relating to operational difficulties such as IT issues.

*“I think the issues we have had with it is legacy that it was clunky*, *you’re having to run a separate software system rather than just use the one pharmacy system that they are currently using … so those have been the disadvantages of it …*” **AHSN employee, Area 1**

Experiences of ease of use boosted motivation for continued engagement as did experiences of usefulness and effectiveness.

*“… I can see it is already having an impact and it will continue to have one going forward as well*. *It is a really positive experience*, *it is definitely something I am going to carry on using going forward within my practice and I think that the idea is fantastic in terms of keeping an eye on patient safety and monitoring …*” **Clinical Pharmacist, Practice 1, Area 3**

The quote below gives an example of a participant’s enthusiasm surrounding positive results and how they showcased this to someone in a senior role who was likely to be in a decision-making position.

*“… when I got the results I showed her [the medical director] because actually that was a significant difference [between PINCER results in June and PINCER results in Oct] … and I think it needs to be shown*, *actually that the amount of work that [Pharmacist 1] and I have put in has actually paid off quite a lot …*” **Practice Pharmacist, Practice 2, Area 3**

The functionality of being able to make contrasts across CCGs and practices received some positive feedback and was also considered to be motivational, regardless of the results.

*“… I was quite looking forward to being able to do the comparison between us and other surgeries … and actually*, *we were quite bad but you know nevertheless it is quite good to look at other surgeries because it also does give you a little bit of a kind of buzz …*” **GP/Prescribing Lead, Practice 4, Area 3**

In contrast, experiencing difficulty of use could reduce motivation or result in discontinuation of use. As one survey respondent stated, they previously used PINCER but no longer do as the “*System was difficult to use in comparison with [other] searches that can be run on SystmOne*.” **Practice T, Area 1 (Respondent–Practice manager)**

*Suggested adaptations*. Evaluations of PINCER also included suggestions on how it could be improved upon in relation to the indicators, including … “*there could be* … *more indicators incorporated into it like going to the future …”* **PCN Pharmacist, Practice 7, Area 3, FU interview (Quote 13, S3 Table).** There were also some intentions of using it as an educational tool for less experienced prescribers. For example, a survey respondent stated that “… *I now actively run the searches regularly*. *I am hoping that with time*, *I will begin to use this as a tool for education sessions with trainees and present findings on a quarterly basis at practice meetings …”* **Practice K, Area 1 (Respondent–PCN Pharmacist).**

## Discussion

This first longitudinal process evaluation of PINCER during the natonal roll-out phase identified novel insights into the variability in awareness, understanding and perceived or experienced benefits and drawbacks of its use.

Inclusion in policy, for instance the incoporation of PINCER in QOF 2019/20 [29] helped clarify misunderstandings which had been problematic amongst the early adopter AHSN regions. Stakeholder investment, the fit with own and organisational goals, incentives and influence of evidence played a role in raising enthusiasm and encouraging uptake. The sources of influential evidence changed throughout the roll-out. At the beginning of the roll-out, a Lancet publication [10] and the evidence-base of the indicators helped to boost credibility, whereas evidence surrounding uptake and success helped drive PINCER forward as the roll-out progressed. Accessibility of troubleshooting support, organisational re-structures, the pandemic and perceptions surrounding workload demands and capacity had an impact on participants’ willingness and ability to adopt and run the intervention within their organisations over time. Variability was also observed in the extent to which participants engaged in operational activities including undertaking training, involving and including others, utilising intervention components and any actions taken towards overcoming barriers or optimising facilitators encountered. Despite experiencing or perceiving difficulties there were signs of successful embedding in practice and motivation to use the intervention even during challenging and unpredictable times. Reflections of use were mainly positive with negative reports mostly relating to operational issues including IT difficulties. Suggested adaptations, such as adding or updating indicators and using PINCER for teaching purposes, which were mainly given in responses from participants working in longer-term use organisations, indicated intentions for continued use.

### Implementation at scale

Achieving widespread uptake of an intervention is often a slow, challenging and work intensive process [31, 32]. Scaling up to reach more participants with similar characteristics and scaling out to reach those with different characteristics have been described as being non-linear, inconsistent processes entailing complex interactions of policy, priorities and contextual factors [33, 34]. In recognition of such difficulties, recommendations for more successful intervention roll-out include: ensuring the intervention is accessible and credible, identifying and supporting those willing to advocate its use, pro-actively interacting with early adopters, using early adopter activity to communicate and promote use amongst others, giving adopters adequate time to implement, experience and embed the intervention and stakeholders should be prepared to lead by example [32]. Our findings highlight how the effort made by stakeholders fits quite closely with these recommendations, for example, AHSN staff putting in extra work during the early stages of the rollout to clarify misunderstandings, ensure difficulties were resolved and using the most up to date, relevant evidence to help drive the intervention forward. Such effort could in part explain successes in terms of widespread adoption and those outlined in the most recent progress report [13].

### Barriers to implementation

In relation to perceived or experienced barriers, it has also been found that lack of awareness or understanding, time restraints and workload concerns have often been given as reasons for not adopting or fully engaging with an intervention [35, 36]. A systematic review conducted by Lau et al [2015] concluded that implementation of interventions can be optimised if the main contextual barriers, which they acknowledged can change over time, are considered and addressed accordingly [37]. During the time this study was conducted, an overarching and unforeseen challenge was the COVID-19 pandemic which confounded pre-existing barriers and became an important contextual factor in discouraging or making adoption and continued use more difficult. In addition to the pandemic impacting on levels of workload and priorities, timing was also an important factor. For example, funding was coming to an end via the AHSNs for training at a time when capacity for training had already been reduced due to the necessary transition from face-to-face to virtual training. No longer requiring the use of PINCER to fulfil the purposes of QOF was also discouraging for some and was a reason given for discontinued use. This fits with existing evidence which suggests that practitioners decisions in relation to delivering quality improvement schemes can be influenced by what is and is not included in QOF, with those relating to current QOF indicators often being prioritised [38]. These are important barriers which would not have been possible to identify in previous PINCER specific evaluations which were conducted pre-pandemic at a time when there had not been the influence of the transient inclusion in QOF and when the funding stream would have covered the duration of the work. In a similar manner as to how stakeholder input could account in part for successes, the impact of the pandemic and timing in some part could account for diminished interest and effort made to engage with PINCER. However, it is also worth noting that there were still signs of motivation and effort made to undertake training, adopt and continue to use PINCER following the declaration of the pandemic.

#### Facilitators for implementation

Facilitators towards intervention implementation identified in this study such as being able to easily understand and use the components, belief in the intervention, feelings of ownership, experiencing the benefits and being a good fit with individual and organisational goals and reforms in practice have also been reflected in the findings of other work [35, 36, 39]. These factors are also thought to operate optimally within a culture that has clear objectives, good teamwork, time for reflection and a vision of how financially incentivised initiatives can be of benefit to patients [39]. Just as timing and alignment with policy posed barriers, there was also evidence of these aspects facilitating the continued use of PINCER. An example of this is the formation of PCNs and the introduction of the PCN Network Contract Directed Enhanced Service (DES) [30] in 2019. There was evidence of PCN pharmacists recognising how PINCER aligned with the PCN DES and their high-risk medication and safety principles which encouraged continued use, with some also actively using PINCER as a tool to assist with SMRs.

#### Strengths & weaknesses

Due to the impact of the pandemic on primary care and there being a pause on non-COVID-19 related studies at certain time points, recruitment of participants for follow-up interviews was challenging. Nationwide recruitment for the survey was also not possible as responses were returned from only eight out of a possible 15 CRN regions. Nevertheless, a total of 148 participants were recruited from 108 different organisations. The data collected also captured interaction with the intervention prior to and after the declaration of the pandemic when workload became unpredictable, motivation and priorities changed and staff had to adapt rapidly to new ways of working [40]. This helped to gain insight into how longer term embedding of PINCER can be supported when the ways in which we are working are changing and are likely to continue to change for the foreseeable future. Overall, the findings are possibly more beneficial at a time when although there have been continuous challenges, there has also been the opportunity for reflection and receptivity to potential improvements in care delivery [41]_._

As we recruited a mix of administrative and clinical staff, it allowed for a holistic overview of acceptability and what type of work or effort had been made by people in different job roles in relation to understanding, adopting and operationalising the intervention. By conducting a survey in addition to interviews, it facilitated the collection of a wider range of opinions and experiences of PINCER as well as offering participants an anonymous platform in which they could feel comfortable disclosing less positive experiences or perceptions they may hold [42]. Furthermore, by using the NPT, it enabled us to gain a comprehensive understanding of the processes involved in why and how participants engaged with PINCER and identify factors that could lead to successful implementation and sustainable use [43]. This included being able to understand the perceived or experienced barriers and facilitators towards gaining an understanding of the intervention and sharing this knowledge with others, making decisions on whether to implement PINCER and continue with its use, working to operationalise it and also how reflecting on its use could also influence further decisions and intentions relating to future use.

### Implications for future research and practice

Being able to balance priorities appeared to be an important aspect involved in the decision-making process surrounding whether to implement PINCER and continue with its use. Factors that influenced these decisions included capacity concerns, current policies, the fit of PINCER with organisational and staff values and the acknowledgment of the importance of medicines optimisation. Further exploratory work could help establish how these aspects could be optimised or overcome as well as gain further insight into how the dynamics between incentives, values, priorities and decisions behind care provision operate.

Delivering new practices or more complex care normally requires co-ordination, collaboration and delegation of tasks both across and within organisations, which if done without appropriate consideration or planning can become fragmented and less effective [44]. Of relevance to this, the recent evaluation of PSI based interventions, including PINCER, highlighted that taking a team approach across the health-care system and professions was deemed important by relevant stakeholders [17]. In regards to within organisation approaches, in this study there was a lack of inclusivity or not taking a more practice-wide approach observed amongst some of the participating organisations. Exploring the mechanisms behind this could help establish how more open communication could be achieved, how teamwork could become more streamlined and actions taken in relation to the intervention be carried out in a more systematic and effective manner both across and within organisations.

There was also the realisation that not all indicators were relevant for different practice demographics which can be problematic for implementation and for intervention designers due to the feasibility of being able to cover all needs [45]. However, in line with some participants reporting that running all thirteen indicators was not possible, being able to be selective with the indicators may be considered beneficial as it could potentially make running the intervention more relevant and manageable. This reflects findings from a process evaluation of a real time prescribing safety dashboard [46] in which it was highlighted that users of the dashboard had a tendency to prioritise the indicators that yielded the highest number of patients identified as being at risk. The findings of the real time prescribing safety dashboard evaluation [46] also showed a tendency towards intensive use following implementation which became less frequent over time. This differs slightly to the findings of this study in the respect that seeing a reduction in numbers of patients identified at risk between running PINCER at one time point to the next appeared to boost motivation for continual and sustained use. Whether this difference is influenced by the intended frequency of use for an intervention or the indicators selected is also an aspect worth exploring.

Some participants reported using other interventions with similar purposes as being a barrier to using PINCER. As there are many other primary care interventions available with more being developed [37], understanding what could maintain the position of PINCER within primary care when policies, agendas and priorities change would be beneficial. In terms of strategies, the National Overprescribing Review Report (2021) [47] advocates that methods for prescription reviews are efficient, technology is used optimally and alternative, more effective medicines should be offered where relevant, which aligns with the functionalities of PINCER. The NHS Long Term Plan [48] also recognises the need to address medication errors in primary care and one of the ways it proposes to do this is by funding more PCN pharmacists to conduct SMRs for patients with long-term health conditions. As highlighted in the results of this study, some PCN pharmacists were already using PINCER as a tool to assist with their SMR work. However, the results also indicated varying ability to differentiate PINCER from other prescribing interventions as well as perceiving some systems as being interchangeable with PINCER, for example, Eclipse (https://www.eclipsesolutions.org/eclipseinfo/abouteclipse/). Having a more centralised, accessible channel in which prescribing safety interventions are clearly defined alongside how they could fit with current policies and strategies, and how they can be used synergistically, where relevant, could help establish the role of PINCER and how it can become an integral part of wider medicines optimisation strategies.

These findings support other work [17] in highlighting the importance of intervention alignment with stakeholder values and local and national agendas, taking a team approach and emphasising how different interventions can be used in a complementary rather than interchangeable manner. Overall, the findings of this study could form the basis of a framework or checklist for the successful implementation and sustainable use for other primary care interventions.

## Conclusions

This study has utilised NPT to advance our understanding of the interplay between individual and contextual factors that determine motives behind implementing and running PINCER in primary care and what can make its use sustainable. Influential drivers for the intervention were commitment and good communication from stakeholders both between one another and down to practice level, peer influence, the influence of the evidence behind the intervention as well as on uptake and effectiveness, inclusion in policy which had an interrelationship with financial incentives and positive experiences of use. Aspects that could further enhance interest and uptake include optimising support available, encouraging a more inclusive and comprehensive approach to use, considering contextual barriers and how they can be overcome and considering timing and fit with organisational and wider agendas. Findings from this study could also be used to help inform uptake and sustainable use of other interventions aimed at preventing avoidable harm or enhancing medicines optimisation in primary care settings.

## Supporting information

S1 Table Details of interviewees. (DOCX)

S2 Table Details of survey respondents. (DOCX)

S3 Table Reference quotes. (DOCX)

S1 Appendix Semi-structured interview templates. (DOCX)

S2 Appendix Download of survey questions. (PDF)
