# Supplementary material for: Understanding factors influencing uptake and sustainable use of the PINCER intervention at scale: A qualitative evaluation using Normalisation Process Theory
Source: PLoS One. 2022 Sep 19;17(9):e0274560. doi: 10.1371/journal.pone.0274560 (PMC9484679; doi:10.1371/journal.pone.0274560)
Supplement: S1 Table — (DOCX) [file pone.0274560.s001.docx]

S1 Table. Details of interviewees*

Areas 1-4 are the same areas for the interviews and survey

| **Establishment type** | **Job role** | **n =** | **Time involved with PINCER** | **Follow-up Interview** |
| --- | --- | --- | --- | --- |
| Higher education | Intervention developer  Personnel responsible for PINCER roll out | 2  3 | >18 months | N  N |
| **Area 1** | | | | |
| AHSN | * | 1 | >18 months | Y |
| CCG 1 | Senior Innovation Project Lead /CCG Pharmacist  Chief Pharmacist | 1  1 | >18 months  >18 months | Y  Y |
| Practice 1 | Clinical Pharmacists | 2 | <6 months (no longer using at time of interview) | N |
| Practice 2 | GP/Research Lead | 1 | >18 months | N |
| Practice 3 | Practice Manager | 1 | between 6–18 months | N |
| Practice 4 | GP Principal  Practice Manager | 1  1 | >18 months | N |
| Practice 5 | Medicines Optimisation Pharmacist | 1 | >18 months | N |
| **Area 2** | | | | |
| AHSN | *  * | 1  1 | >18 months  >18 months | Y  Y |
| CCG1 | Locality Lead Pharmacist  Medicines Optimisation Technician | 1  1 | >18 months  >18 months | N  Y |
| Practice 1 | Practice Manager | 1 | >18 months | N |
| Practice 2 | Practice Manager/Practice Nurse  Lead Dispenser  Medical Secretary | 1  1  1 | >18 months  >18 months  >18 months | N  N  N |
| Practice 3 | Advanced Nurse Practitioner  Medicines Manager | 1  1 | >18 months  >18 months | N  N |
| Practice 4 | GP/Prescribing Lead | 1 | >18 months | N |
| Practice 5 | GP/QoF lead  Data Lead | 1  1 | >18 months  >18 months | Y  Y |
| **Area 3** | | | | |
| AHSN | * | 1 | between 6-18 months | Y |
| CCG 1 | CCG Prescribing Support Pharmacist | 1 | >18 months | Y |
| Practice 1 | Clinical Pharmacist | 1 | between 6-18 months | Y |
| Practice 2 | GP  Clinical Pharmacist  Practice Pharmacist | 1  1  1 | between 6-18 months  between 6-18 months  between 6-18 months | Y  Y  Y |
| Practice 3 | Practice Manager  Lead Dispenser | 1  1 | between 6-18 months  between 6-18 months | N  N |
| Practice 4 | GP/Prescribing Lead  Lead Clinical Pharmacist  Practice Pharmacist | 1  1  1 | between 6-18 months  between 6-18 months  between 6-18 months | N  Y  Y |
| Practice 5 | GP  Clinical Pharmacist | 1  1 | between 6-18 months  between 6-18 months | N  N |
| Practice 6 | GP  Clinical Pharmacist  PCN Pharmacist | 1  1  1 | >18 months  >18 months  >18 months | N  N  N |
| Practice 7 | GP  PCN Pharmacist | 1  1 | between 6-18 months  between 6-18 months | N  Y |
| **Area 4** | | | | |
| AHSN | * | 1 | between 6-18 months | N |
| CCG 1 | Pharmacy Technician | 1 | between 6-18 months | Y |
| Practice 1 | Practice Nurse  Practice Pharmacist | 1  1 | >18 months  >18 months | N  N |

*Job titles of AHSN staff have been withheld due to the possibility of participant identification
