# Supplementary material for: Understanding factors influencing uptake and sustainable use of the PINCER intervention at scale: A qualitative evaluation using Normalisation Process Theory
Source: PLoS One. 2022 Sep 19;17(9):e0274560. doi: 10.1371/journal.pone.0274560 (PMC9484679; doi:10.1371/journal.pone.0274560)
Supplement: S2 Table — (DOCX) [file pone.0274560.s002.docx]

S2 Table. Details of survey respondents

Areas 1-4 are the same areas for the interviews and survey

| **Establishment type** | **Job role** | **n =** | **Time involved with PINCER** | **Current user** |
| --- | --- | --- | --- | --- |
| **Area 1** | | | | |
| CCG A | CCG Pharmacist | 1 | >18 months | Y |
| Practice A | GP | 1 | <6 months | N – previous user |
| Practice B | Practice Manager | 1 | >18 months | Y |
| Practice C | CCG Pharmacist | 1 | >18 months | Y |
| Practice D | GP | 1 | <6 months | N – previous user |
| Practice E | Practice Nurse | 1 | between 6-18 months | Y |
| Practice F | Practice Pharmacist | 1 | >18 months | Y |
| Practice G | PCN Pharmacist | 1 | >18 months | Y |
| Practice H | CCG Pharmacist  Practice Pharmacist | 1  1 | >18 months | Y |
| Practice I | Practice Manager | 1 | >18 months | Y |
| Practice J | Practice Manager | 1 | between 6-18 months | N – previous user |
| Practice K | PCN Pharmacist | 1 | >18 months | Y |
| Practice L | CCG Pharmacist | 1 | >18 months | Y |
| Practice M | Practice Pharmacist  PCN Pharmacist | 1  1 | between 6-18 months | N – previous user |
| Practice N | PCN Pharmacist | 1 | >18 months | Y |
| Practice O | Practice Pharmacist | 1 | between 6-18 months | Y |
| Practice P | PCN Pharmacist | 1 | >18 months | Y |
| Practice Q | GP | 1 | >18 months | Y |
| Practice R | CCG Pharmacist | 1 | >18 months | Y |
| Practice S | Deputy Practice Manager | 1 | between 6-18 months | Y |
| Practice T | Practice Manager | 1 | >18 months | N – previous user |
| Practice U | GP | 1 | <6 months | Y |
| Practice V | Practice Manager | 1 | >18 months | Y |
| Practice W | PCN Pharmacist | 1 | >18 months | Y |
| Practice X | Practice Manager | 1 | not reported | N – previous user |
| Practice Y | PCN Pharmacist | 1 | >18 months | N – previous user |
| Practice Z | GP | 1 | >18 months | Y |
| Practice A1 | Practice Pharmacist | 1 | >18 months | Y |
| **Area 2** | | | | |
| CCG A | CCG Pharmacist | 1 | >18 months | Y |
| Practice A | Practice Manager | 1 | >18 months | Y |
| Practice B | PCN Pharmacist | 1 | between 6-18 months | Y |
| Practice C | GP | 1 | >18 months | Y |
| Practice D | GP | 1 | >18 months | Y |
| Practice E | CCG Pharmacist | 1 | >18 months | Y |
| Practice F | GP | 1 | >18 months | Y |
| **Area 3** | | | | |
| Practice A | PCN Pharmacist | 1 | >18 months | Y |
| Practice B | PCN Pharmacist | 1 | between 6-18 months | Y |
| Practice C | Practice Pharmacist | 1 | between 6-18 months | Y |
| Practice D | Clinical Pharmacist | 1 | never used | N |
| Practice E | GP | 1 | >18 months | Y |
| **Area 4** | | | | |
| CCG A | CCG Pharmacy Technician | 1 | <6 months | N – previous user |
| Practice A | Practice Pharmacist | 1 | >18 months | N – previous user |
| Practice B | Practice Pharmacist  Practice Nurse | 1  1 | >18 months | Y |
| **Area 5** | | | | |
| Practice A | PCN Pharmacist | 1 | between 6-18 months | Y |
| Practice B | Practice Pharmacist  PCN Pharmacist | 1  1 | between 6-18 months | Y |
| **Area 6** | | | | |
| Practice A | Practice Manager  GP | 1  1 | never used | N |
| Practice B | Practice Manager  PCN Pharmacist | 1  1 | never used | N |
| Practice C | GP | 1 | <6 months | Y |
| Practice D | Practice pharmacist | 1 | between 6-18 months | N – previous user |
| Practice E | GP | 1 | <6 months | Y |
| Practice F | PCN Pharmacist | 1 | between 6-18 months | Y |
| Practice G | Practice Manager | 1 | between 6-18 months | Y |
| Practice H | GP | 1 | between 6-18 months | Y |
| Practice I | Administrator  Practice Manager  Lead GP  PCN Pharmacist | 1  1  1  1 | <6 months | Y |
| Practice J | PCN Pharmacist  Practice Manager | 1  1 | between 6-18 months | Y |
| Practice K | Practice Pharmacist | 1 | between 6-18 months | Y |
| Practice L | Practice Manager  PCN Pharmacist | 1  1 | between 6-18 months | Y |
| Practice M | GP | 1 | never used | N |
| Practice N | Practice Pharmacist | 1 | between 6-18 months | N – previous user |
| Practice O | GP | 1 | >18 months | Y |
| Practice P | PCN Pharmacist | 1 | never used | N |
| Practice Q | Practice Manager | 1 | between 6-18 months | N – previous user |
| Practice R | GP | 1 | between 6-18 months | N – previous user |
| Practice S | Practice Pharmacist | 1 | <6 months | Y |
| Practice T | GP | 2 | between 6-18 months | Y |
| Practice U | Practice Nurse | 1 | <6 months | Y |
| Practice V | Practice Pharmacist  Clinical Lead GP | 1  1 | <6 months | Y |
| Practice W | GP  Practice Manager  PCN Pharmacist | 1  1  1 | >18 months | N – previous user |
| Practice X | Practice Pharmacist  PCN Pharmacy Technician | 1  1 | <6 months | N – previous user |
| Practice Y | Practice Pharmacist | 1 | between 6-18 months | Y |
| Practice Z | Administrator | 1 | never used | N |
| Practice A1 | Senior Manager | 1 | >18 months | Y |
| Practice B1 | Practice Manager | 1 | never used | N |
| Practice C1 | PCN Pharmacist | 1 | between 6-18 months | Y |
| Practice D1 | GP | 1 | between 6-18 months | Y |
| Practice E1 | GP | 1 | never used | N |
| Practice F1 | GP | 1 | never used | N |
| Practice G1 | Practice Pharmacist | 1 | between 6-18 months | Y |
| Practice H1 | PCN Pharmacist | 1 | between 6-18 months | Y |
| Practice I1 | Practice Manger | 1 | between 6-18 months | Y |
| **Area 7** | | | | |
| Practice A | PCN Pharmacist | 1 | between 6-18 months | Y |
| **Area 8** | | | | |
| Practice A | Practice Manager | 1 | >18 months | Y |
| Practice B | PCN Pharmacist | 2 | between 6-18 months | Y |
| Practice C | Practice Pharmacist | 1 | >18 months | Y |
