# Supplementary material for: Understanding factors influencing uptake and sustainable use of the PINCER intervention at scale: A qualitative evaluation using Normalisation Process Theory
Source: PLoS One. 2022 Sep 19;17(9):e0274560. doi: 10.1371/journal.pone.0274560 (PMC9484679; doi:10.1371/journal.pone.0274560)
Supplement: S3 Table — (DOCX) [file pone.0274560.s003.docx]

S3 Table. Reference quotes

| **Coherence - Awareness & perceptions** | |
| --- | --- |
| Understanding of the PINCER intervention | |
| Quote 1 | *“… and obviously it going in to QOF and … the Patient Safety Strategy, those things were enormously helpful … I think the other thing that was really helpful in policy terms was the World Health Organization Global Challenge because that talks about avoidable medication errors which is exactly what PINCER finds, so that … having that as you know with the ambiguity that was happening in England a little bit, having the World Health Organization statement over the top, that was also kind of helpful.”* **AHSN employee, Area 2** |
| **Cognitive Participation** | |
| Stakeholder interaction | |
| Quote 2 | *“There have been some dark days but you know we get on well as a group and we just kept talking and that has been really important … and I think what we were able to do as [personnel responsible for the roll out] to us, myself and [colleague’s name] from [Area 2] AHSN was just keep going together … We worked really, really hard to keep the show on the road.”* **AHSN employee, Area 2** |
| Quote 3 | *“The CCG’s … [were] sort of drip feeding evidence, information, … getting buy in from workshops … that we used to sort of have a detailed conversation round it, for GPs it was much more sort of giving them the sort of high level information, what is PINCER, how can it benefit you, what you need to do to be able to sign up you know pretty sort of basic stuff … obviously with all of it we try and sort of include the key bits of evidence and references and so forth but yes it has been mostly sort you know cascaded information …”* **AHSN employee, Area 3** |
| Influence of evidence | |
| Quote 4 | *“… so I think the biggest thing that has happened in the last 12 months, obviously COVID-19 aside, is that we got the beginnings of the data from the interim report … That has been a game changer because instead of us talking about a study that was published in The Lancet in 2012, and some local data … we’re now talking about you know nationally over 1,060 practices have completed the process. So what that does straight away is it gets rid of the arguments about oh it is too difficult, we can’t do it, over 1,000 practices have done it and we have got that [data] at patient level … we know what happened before, we know what happened afterwards and that has been hugely, hugely powerful.”* **AHSN, employee, Area 2, Follow-up interview** |
| *Incentives and inclusion in policy* | |
| Quote 5 | *“You don’t have to [go with what the CCG drive forward] but if you want their payments then you know, you can opt out because you have always got a choice but you know I like to make money for the practice so yes keep it coming …”* **Practice Manager, Practice 3, Area 3** |
| Capacity and contextual factors influencing decisions to adopt and use PINCER | |
| Quote 6 | *“Large practice size of over 20,000 patients and 10 GPs, only one clinical pharmacist at present. Waiting for the Pharmacy Team to expand before utilising PINCER. Other medication safety programmes such as Eclipse are in place to assist with medication monitoring for now. PINCER was also not considered a priority as lack of access to phlebotomy services during the COVID-19 pandemic, hence, monitoring of key medication lines such as Aceis [Angiotensin-converting inhibitors] and ARBs [angiotensin-receptor blockers] is not taking place for now.”* **Practice D, Area 3 (Respondent – Clinical Pharmacist)** |
| *‘*Fit’ with own and organisational objectives and values | |
| Quote 7 | *“So, in principle it is a patient safety tool, what does it mean for me as a clinician? It just means extra work … given a list of patients that you know someone has landed on my lap, if I don’t deal with them, they come to harm then they say well it was on your lap, so it feels like there is extra work, I understand the principle behind it, I understand the benefits behind it you know [but] in a very busy working life it is … another bit of work you have to squeeze in somewhere …”* ***GP,* Practice 2, Area 3** |
| **Collective Action** | |
| Implementing and running PINCER | |
| Quote 8 | *“So, I think we have got five practices that deal with [clinical system X] and we were planning to use the PINCER searches to give us the numbers of patients at the risk of GI bleed and then show an improvement after having done the work. But we have not managed to get that running because of … well to be fair I have tried to run it a few times but I wasn’t able to make it work through [the upgraded version of clinical system X] .. So, I haven’t managed to make it run yet, and then obviously with the vaccine, things have taken over ever since ...”* **CCG Prescribing Support Pharmacist, CCG1, Area 3, FU interview** |
| Quote 9 | *“… you do need to actually set aside time to just sit down not interrupted and actually kind of go right, I am going to … you then need to think about the structure of what you’re going to do … to make sure that this carries on working going forward …”* **GP/Prescribing Lead, Practice 4, Area 3** |
| Quote 10 | *“… at the moment the main one that we focus on like I said is indicator E2 [prescription of aspirin in combination with another antiplatelet drug (without co-prescription of an ulcer-healing drug)] … that was one that we went into detail on that was actually done as a full in depth root cause analysis, it was presented as a PowerPoint presentation at one of our GP meetings, and then … the sort of end of it was left open to discussion with all clinical colleagues so with a view to devising an action plan as to how we were going to help these patients essentially and make changes within our system in practice, so the idea going forward now is that we are going to do that for each and every one of the indicators …”…* **Clinical Pharmacist, Practice 1, Area 3** |
| Signs of embedding and commitment | |
| Quote 11 | *“… we have put alerts on …” [Res 1] “Oh yes so when you prescribe an NSAID [non-steroidal anti-inflammatory drug] now … if you type in a shortcut like instead of Ibuprofen, if you type in IBU it creates a shortcut that would then say … take one tablet three times a day, alongside Omeprazole to protect your stomach so there is an automatic reminder there…” [Res 2***] Data lead & GP, Practice 5, Area 2** |
| Quote 12 | *“A Clinical letter [was] created for medicines reconciliation to obtain missing or unclear information regarding antiplatelets or anticoagulation … [and] … Used as needed”* **Practice A, Area 4 (Respondent – Practice Pharmacist)** |
| **Reflexive Monitoring** | |
| Suggested adaptations | |
| Quote 13 | *“I do think for the PINCER indicators there could be more indicators, more indicators incorporated into it like going to the future … you could have it in sub-sections … And then in each sub-section have like even more sub-sections … for like the NSAIDs and then a section for cortical steroids.”* **PCN Pharmacist, Practice 7, Area 3, FU interview** |
