# Supplementary material for: Understanding factors influencing uptake and sustainable use of the PINCER intervention at scale: A qualitative evaluation using Normalisation Process Theory
Source: PLoS One. 2022 Sep 19;17(9):e0274560. doi: 10.1371/journal.pone.0274560 (PMC9484679; doi:10.1371/journal.pone.0274560)
Supplement: S1 Appendix — (DOCX) [file pone.0274560.s004.docx]

S1 Appendix. Semi-structured interview templates

**Practice and CCG Staff and Stakeholder Interview Schedule**

**OPENING**

- Check if participant has read the information sheet and if they have any questions
- Complete/reiterate consent and confidentiality issues
- Discuss ‘ground rules' for interviews conducted as a group
- *We are currently conducting a process evalutation to understand what factors influence the sustained implementation and operation of the PINCER intervention in primary care. This understanding will enable us to provide recommendations for wider roll-out of PINCER in general practice. As part of this interview we will be asking you to share your thoughts and experiences of PINCER in your working role.*

**Background information:**

- May you tell me a little bit about your professional background and current place(s) of work? *Profession/job title? Years of experience?*
- How did you hear about PINCER? *Where did you hear about it? When did you hear about it?*
- How would you describe the PINCER intervention? *What have you heard/been told about PINCER?*
- What is your current interaction/role with PINCER? *How has this changed over time/since the last interview?*
- How long has the PINCER intervention been used in your workplace? *At what point did you become involved with the PINCER intervention? Length of time involved with PINCER?*

**Main discussion:**

- How was the PINCER intervention introduced (or continued) to be used in your workplace*?*
- Who were/are the key influential individuals (prompt for roles) in driving the PINCER intervention forward? *Why influential – endorsement, support (resources, training, financial), involvement?* Are they able and willing to get others involved in PINCER?
- What is/was the purpose of using the PINCER intervention in your practice? *What are/were you hoping to achieve? What is it there for? How should it be used? Do other staff at your workplace share this view?*
- What training have you received regarding your use of the PINCER intervention? *Where trained? When trained? Is sufficient training provided to enable staff to use the PINCER intervention?*
- How is the PINCER process carried out/run in your CCG/practice? *Identifying patients (queries & list)? Sharing list with GP/GP involvement? Acting on results (individual patient and/or process)?*
- What reports do you use / are you aware of at present how PINCER intervention has worked in your workplace? How useful do you find these *– understood, timely, relevant, useful?* A*re you seeing any improvements? Why? How do you use these in your CCG/Practice? If states not useful/didn’t understand – did they seek clarification/help?*
- Do you have any ways in which the team get together to talk about the PINCER intervention and how well it is working for you? *Who is responsible for driving/organising this and what have been the outputs of such activity?*
- How confident are you using the PINCER intervention? *Reason for level of confidence? Has this changed over time? And if so why? Confidence that others are able to use PINCER?*
- Are the PINCER tasks allocated to those with the right mix of skills and training to undertake PINCER tasks?
- What challenges have you faced with using PINCER? *How resolved?* What has gone well? *Why?*
- Have you been able to make any modifications with how you use the PINCER intervention in practice? *What are these modifications?*
- Are there modifications you would like to make that you have not been able to make? *Why?*
- Are there components/aspects of PINCER that should not be altered?

PINCER intervention comprises the following:

- Practice staff conducting searches on GP computer systems using MIQUEST software to identify patients at risk of a range of common and important prescribing and drug monitoring errors;
- Pharmacists, specifically trained to deliver the intervention, meeting with GPs and nurse prescribers in each general practice to: (a) Discuss the results of the computer searches, and use educational outreach techniques to highlight the importance of the prescribing errors identified; (b) Agree an action plan for correcting the errors identified and improving safety systems for prescribing and monitoring of medications using the principles of root cause analysis;
- These pharmacists (and pharmacy technicians) working with general practice staff (doctors, nurses and administrators) to institute the agreed action plan. This includes a range of approaches such as inviting patients into the surgery to discuss medication changes and to have essential blood tests done, and improving prescribing safety systems within practices to prevent future problems.
- What are the potential values, benefits and importance of the PINCER intervention to your work?
- How different is the PINCER intervention from your usual ways of working?
- How well does PINCER fit with existing role? *Has PINCER become an integral part of your daily work?* *Easy or hard to integrate?*
- Has PINCER provided new/different ways of working with your colleagues? What are some examples? *Strengthened or disrupted working relationships?*
- Has PINCER provided new/different ways of interacting and communicating with your patients? *How have you communicated PINCER with your patients? Strengthened or disrupted patient/practitioner relationships?*
- Do you believe that participating in PINCER is appropriate for (i) your job/your role and (ii) use in everyday practice across primary care?
- Do you value the effects that the PINCER intervention has had on your work?
- What is the view of other staff whether it is worthwhile using PINCER or not?
- Will you and/or workplace/organisation continue to support the ongoing use of the PINCER intervention? *What role will you be playing? Are there any other roles you are planning to undertake?*
- What resources and support (management level, policy level, national level) do you need if PINCER is going to continue to be used in your workplace?

**CLOSING**

- Is there anything else you wish to discuss about the roll out of the PINCER intervention
- Any suggestions of others (person or practice) you think we should interview?
- Mention what happens next: transcribing, analysis, and summarising findings to help improve PINCER roll-out
- Remind participant that if they have any questions/queries that crop up they can be in touch
- Thank you!

**Non-Practice & CCG Staff and Stakeholder Interview Schedule**

**OPENING**

- Check if participant has read the information sheet and if they have any questions
- Complete/reiterate consent and confidentiality issues
- Discuss ‘ground rules' for interviews conducted as a group
- *We are currently conducting a process evalutation to understand what factors influence the sustained implementation and operation of the PINCER intervention in primary care. This understanding will enable us to provide recommendations for wider roll-out of PINCER in general practice. As part of this interview we will be asking you to share your thoughts and experiences of PINCER in your working role.*

**Background information:**

- Can you tell me a little bit about your professional background and current place(s) of work? *Profession/job title? Years of experience?*
- How long has the PINCER intervention been used in your organisation? *Your role in PINCER? At what point did you become involved with the PINCER intervention? Length of time involved with PINCER?*
- What is your current interaction/role with PINCER? *How has this changed over time/since the last interview?*

**Main discussion:**

[NB. Not all of these questions are relevant for all participants, and the field researcher will need to determine which questions are appropriate based upon the participant’s role and background]

**Overview:**

- Can you describe the role that you/your organisation have had in the development/implementation of PINCER? Describe the timeline where possible.

**The intervention**:

- What is its purpose or contribution to safer prescribing?

(If not already covered above) Can you describe the timeline or life cycle of the intervention from conception through to adoption? (The following questions tease out element of the timeline, or of the participant cannot do the timeline, these will help with exploring different issues)

- How and when was it developed?
- Did a particular group or organisation play a significant role in its development; if so, how?
- How has it been through significant iterations and revisions, why?
- What is the evidence-base (who developed it and where was it published)?
- How was the intervention first tested and developed in practice settings?

**The spread and adoption:**

- How has the intervention been communicated and disseminated across the NHS?
- What role have policy and service leaders had supporting spread and adoption?
- How have NHS, commercial or other agencies supported the use of the intervention?
- Has there been any reticence or resistance to adopting the intervention, how were these mediated?
- What have been the main push and pull factors influencing adoption and spread?
- How has the intervention aligned with or benefited from other policy developments, media issues or other service priorities?

**The future**:

- What do you think would support the future long-term embedding of the intervention?

**CLOSING**

- Is there anything else you wish to discuss about the roll out of the PINCER intervention
- Any suggestions of others (person or practice) you think we should interview?
- Mention what happens next: transcribing, analysis, and summarising findings to help improve PINCER roll-out
- Remind participant that if they have any questions/queries that crop up they can be in touch
- Thank you!
