# Supplementary material for: Understanding factors influencing uptake and sustainable use of the PINCER intervention at scale: A qualitative evaluation using Normalisation Process Theory
Source: PLoS One. 2022 Sep 19;17(9):e0274560. doi: 10.1371/journal.pone.0274560 (PMC9484679; doi:10.1371/journal.pone.0274560)
Supplement: S2 Appendix — (PDF) [file pone.0274560.s005.pdf]

Process Evaluation of PINCER - PRoTeCT Staff Questionnaire

Taking part in our research

The PINCER intervention comprises of a number of key components that are organised to facilitate searching GP computer systems to identify patients who are potentially at on-going risk from their prescriptions, and then acting to correct the problem.

We are interested in understanding your experiences of using PINCER in primary care, over time, so we can understand how to improve further use and its wider adoption.

Please help us by taking time to complete this questionnaire. This questionnaire can be completed by those working within general practice and/or clinical commissioning groups (CCGs).

We ask that someone working at your practice/s or CCG with a good understanding of how PINCER was implemented and used over time completes this questionnaire. Please collaborate with your colleagues to help you answer all the questions where you feel this is needed.

This questionnaire will take around 10 - 20 minutes to complete**.** All information you provide will be kept strictly confidential.

If you have any questions or wish to clarify any information before completing the questionnaire please contact us using the contact details provided in the participant information sheet.

I have read the information sheet, version xx, dated xx and give my consent to take part. ✱ *Required*

- ○ Yes
- ○ No

The PINCER intervention

Are you are completing this questionnaire on behalf of a general practice/s or a CCG? **(n.b. If you are a PCN or CCG employee but have a patient facing role in a general practice or practices, please select the general practice option. If however, you work predominately at CCG level, please select the CCG option)**

The PINCER intervention - questions for staff working within general practices

Please answer the following questions relating to the use of the PINCER intervention within your general practice/s.

Please note, throughout the questionnaire:

- **when providing a free text response, each text box can be expanded by dragging the bottom right hand corner with your cursor.**
- **you are not required to put a response in every row for questions that are in table format.**

Does your general practice/s currently use the PINCER intervention?

- ○ Yes
- ○ No, but has used it previously
- ○ No

If you answered 'Yes', could you please tell us why your practice/s are currently using PINCER.

If your practice/s has used PINCER previously but does not use it currently, please state why below.

Please provide details below in relation to any PINCER training activity(ies) undertaken at your practice/s, **using a separate line for each activity**. (If there has been/are none please state 'None' in the first text box).

	Please describe the activity(ies) below using a separate line for each one.	Which professional role/s (if known) were/are responsible for these activities?	If you selected Other, please specify:
1		Please select	
2		Please select	
3		Please select	
4		Please select	
5		Please select	
6		Please select	

Was anything done by you/your practice/s to highlight the PINCER intervention to patients and/or carers registered with your general practice/s?

- Yes
- No

If your answer was 'Yes', please give details of what this was:

If your answer was 'No', please state why not:

Who, as part of the PINCER intervention, conducts/conducted searches on your general practice/s computer systems to identify patients at risk of a range of common and important prescribing and drug monitoring errors? Please select a box for each professional group below. *Optional*

Please don't select more than 1 answer(s) per row.

	Never	Rarely	Sometimes	Usually	Always	Not applicable (i.e. job role does not exist in practice/s)	I don't know
General practitioner/s	☐	☐	☐	☐	☐	☐	☐
Practice Nurse/s/Advance Nurse Practitioner/s	☐	☐	☐	☐	☐	☐	☐
Practice Manager	☐	☐	☐	☐	☐	☐	☐
Administrator/s	☐	☐	☐	☐	☐	☐	☐
Data Lead/s	☐	☐	☐	☐	☐	☐	☐
PCN Pharmacist/s	☐	☐	☐	☐	☐	☐	☐
Practice-based Pharmacist/s	☐	☐	☐	☐	☐	☐	☐
PCN Pharmacy Technician/s	☐	☐	☐	☐	☐	☐	☐
Practice-based Pharmacy Technician	☐	☐	☐	☐	☐	☐	☐
Dispenser/s (in dispensing practices)	☐	☐	☐	☐	☐	☐	☐
CCG Pharmacist/s	☐	☐	☐	☐	☐	☐	☐
CCG Pharmacy Technician/s	☐	☐	☐	☐	☐	☐	☐
Other	☐	☐	☐	☐	☐	☐	☐

Please specify the job role/s for 'Other' where applicable:

Please select which staff member/s have/had a lead role in in communicating the findings generated by the PINCER electronic health record searches to the other staff members?

Please don't select more than 1 answer(s) per row.

	Never	Rarely	Sometimes	Usually	Always	Not applicable (i.e. job role does not exist in practice/s)	I don't know
General practitioner/s	☐	☐	☐	☐	☐	☐	☐
Practice Nurse/s/Advance Nurse Practitioner/s	☐	☐	☐	☐	☐	☐	☐
Practice Manager	☐	☐	☐	☐	☐	☐	☐
Administrator/s	☐	☐	☐	☐	☐	☐	☐
Data Lead/s	☐	☐	☐	☐	☐	☐	☐
PCN Pharmacist/s	☐	☐	☐	☐	☐	☐	☐
Practice-based Pharmacist/s	☐	☐	☐	☐	☐	☐	☐
PCN Pharmacy Technician/s	☐	☐	☐	☐	☐	☐	☐
Practice-based Pharmacy Technician/s	☐	☐	☐	☐	☐	☐	☐
Dispenser/s (in dispensing practices)	☐	☐	☐	☐	☐	☐	☐
CCG Pharmacist/s	☐	☐	☐	☐	☐	☐	☐
CCG Pharmacy Technician/s	☐	☐	☐	☐	☐	☐	☐

Other	☐	☐	☐	☐	☐	☐	☐

Please specify the job role/s for 'Other' where applicable:

Please provide details below of any activity(ies) that take/have taken place to address patients identified as being 'at risk' from their prescription as part of the PINCER intervention, **using a separate line for each activity.**

	Please describe the activity here	Please state how often this activity occurs	General practitioner/s	Practice Nurse/s/Advance Nurse Practitioner/s	Practice Manager	Adm
1			☐	☐	☐	
2			☐	☐	☐	
3			☐	☐	☐	
4			☐	☐	☐	
5			☐	☐	☐	
6			☐	☐	☐	
7			☐	☐	☐	
8			☐	☐	☐	
9			☐	☐	☐	
10			☐	☐	☐	

How long has the PINCER intervention been active/running at your general practice/s?

○ Less than 6 months

- Between 6 - 18 months
- More than 18 months

Have any changes been made to the way you have delivered the PINCER intervention in your general practice/s over time?

- No
- Yes

If your answer was 'Yes', please give details of these changes including reason/s why these changes were made (Please include any impact that the recent COVID-19 pandemic may have had on the delivery of PINCER).

Please tell us why your practice/s does not use PINCER intervention in the text box below.

About you

Please state which CCG your practice/s belong to.

Please complete on behalf of each person involved in completing the questionnaire, using one line per person. **(n.b. if your practice has merged with another or changed its name, please include in your answer all the time you have worked at this practice and its predecessors).**

	What is your (and any other individual/s who has been involved in completing the questionnaire) job role?	If you selected Other, please specify:	How many years have you worked for this practice/s?
1	Please select		Please select
2	Please select		Please select
3	Please select		Please select
4	Please select		Please select
5	Please select		Please select

If you would like to be informed of the results of this survey, please provide your email address/es here.

If you have any additional comments in relation to the PINCER intervention, please add them below.

The PINCER intervention - questions for CCG staff

Please answer the following questions relating to the use of the PINCER intervention within your CCG.

Please note, throughout the questionnaire:

- **when providing a free text response, each text box can be expanded by dragging the bottom right hand corner with your cursor.**
- **you are not required to put a response in every row for questions that are in table format.**

Does your CCG currently use the PINCER intervention? ✱ *Required*

- ○ Yes
- ○ No, but has used it previously
- ○ No

If your answered 'Yes', please give details of the extent of adoption within the CCG and how it is/was being used.

If your CCG has used PINCER previously but does not use it currently, please state why below.

Please provide details below in relation to any PINCER training activity(ies) undertaken at your CCG, **using a separate line for each activity**. (If there has been/are none please state 'None' in the first text box).

	Please describe the activity here	Please specify which professional role/s was/is involved in leading this activity	Please specify which organisation/s was/is involved in leading this activity	If you selected Other, plea
1			Please select	
2			Please select	
3			Please select	
4			Please select	
5			Please select	
6			Please select	

Was anything done to highlight the PINCER intervention to patients and/or carers registered with the general practices of your CCG?

- Yes
- No

If your answer was 'Yes', please give details of what this was below.

If your answer was 'No', please state why not below.

Please state which professional group/s, if any, has/have a lead role **at CCG level** in accessing and using PINCER data on patients at risk from their medication across general practices in your region. (If no professional group/s has/have a lead role, please specify why this is the case)

Please provide details below of any activity(ies) that take/have taken place at CCG level in response to PINCER data highlighting patients at potential risk of harm from their medications across general practices in your region. **Use a separate line for each activity.**

	Please describe the activity here	Please state how often this activity occurs	Please state which professional role/s are/were involved in this activity
1			
2			
3			
4			
5			
6			
7			
8			
9			
10			

Which professional group/s, as part of the PINCER intervention, has most commonly had a lead role **at general practice level** in responding to the data concerning patients potentially at risk from their medications generated by the PINCER electronic health record searches? Please select a box for each professional group below, using your knowledge of PINCER activity across general practices in your region.

Please don't select more than 1 answer(s) per row.

	Never	Rarely	Sometimes	Usually	Always	Not applicable (i.e. job role does not exist in practice/s)	I don't know
General practitioner/s	☐	☐	☐	☐	☐	☐	☐
Practice Nurse/s/Advance Nurse Practitioner	☐	☐	☐	☐	☐	☐	☐
Practice Manager	☐	☐	☐	☐	☐	☐	☐
Administrator/s	☐	☐	☐	☐	☐	☐	☐

Data Lead/s	☐	☐	☐	☐	☐	☐	☐
PCN Pharmacist/s	☐	☐	☐	☐	☐	☐	☐
Practice-based Pharmacist/s	☐	☐	☐	☐	☐	☐	☐
PCN Pharmacy Technician/s	☐	☐	☐	☐	☐	☐	☐
Practice-based Pharmacy Technician/s	☐	☐	☐	☐	☐	☐	☐
Dispenser/s (in dispensing practices)	☐	☐	☐	☐	☐	☐	☐
CCG Pharmacist/s	☐	☐	☐	☐	☐	☐	☐
CCG Pharmacy Technician/s	☐	☐	☐	☐	☐	☐	☐
Other	☐	☐	☐	☐	☐	☐	☐

Please specify the job role/s for 'Other' where applicable:

Changes to routine ways of working can be made within general practices in response to PINCER data. Where such changes have been made across the general practices within your CCG, please provide details of any activity that takes place between the CCG and general practices in relation to this, including if this activity varies between the practices.

Please provide details of any changes that have been made to how the PINCER intervention is delivered within your CCG over time, **using a separare line for each change**. Please include any impact that the recent COVID-19 pandemic may have had.

	Please describe the change here including any related activity(ies)	Please provide the reason for the change	How much time was spent on this change and the relating activity(ies)?
1			
2			
3			
4			
5			
6			

Did patients and carers have any role/contribution in delivering and/or shaping the PINCER intervention within the practices of you CCG?

- ○ No
- ○ Yes

If you answered 'Yes' please provide details of this role/contribution here. Please include any impact that the COVID-19 pandemic may have had.

Please tell us why your CCG does not use PINCER in the text box below.

About you

Please select which AHSN/s your CCG is associated with

- ☐ East Midlands
- ☐ Eastern
- ☐ Health Innovation Manchester
- ☐ Health Innovation Network South London
- ☐ Imperial College Health Partners
- ☐ Innovation Agency
- ☐ Kent, Surrey and Sussex
- ☐ North East and North Cumbria
- ☐ Oxford
- ☐ South West Pennisula
- ☐ University College London Partners
- ☐ Wessex
- ☐ West Midlands
- ☐ West of England
- ☐ Yorkshire and Humber

Please complete on behalf of each person involved in completing the questionnaire. **(n.b. if your CCG has merged with another or changed its name, please include in your answer all the time you have worked at this CCG and its predecessors).**

	What is your (and any other individual/s who has been involved in completing the questionnaire) job role?	If you selected Other, please specify:	How many years have you worked for this CCG?
1	Please select		Please select
2	Please select		Please select
3	Please select		Please select
4	Please select		Please select
5	Please select		Please select

If you would like to be informed of the results of this survey, please provide your email address/es here.

If you have any additional comments in relation to the PINCER intervention, please add them below.

Final page

Thank you for taking the time to complete this questionnaire. Your help is much appreciated.

Key for selection options

2 - Are you are completing this questionnaire on behalf of a general practice/s or a CCG? (n.b. If you are a PCN or CCG employee but have a patient facing role in a general practice or practices, please select the general practice option. If however, you work predominately at CCG level, please select the CCG option)

A general practice/s (including PCN employees)
A clinical commissioning group (CCG)

4.1.b - Which professional role/s (if known) were/are responsible for these activities?

General practitioner/s
Practice Nurse/s/Advance Nurse Practitioner/s
Practice Manager
Administrator/s
Data lead/s
PCN Pharmacist/s
Practice-based Pharmacist/s
PCN Pharmacy Technician/s
Practice-based Pharmacy Technician
Dispenser/s (in dispensing practices)
CCG Pharmacist/s
CCG Pharmacy Technician/s
I don't know
Other

4.1.d - Which organisation/s were/are responsible for these activities

AHSN
CCG
PRIMIS
The practice/s
I don't know
Other

4.2.b - Which professional role/s (if known) were/are responsible for these activities?

General practitioner/s
Practice Nurse/s/Advance Nurse Practitioner/s
Practice Manager
Administrator/s
Data lead/s
PCN Pharmacist/s
Practice-based Pharmacist/s
PCN Pharmacy Technician/s
Practice-based Pharmacy Technician
Dispenser/s (in dispensing practices)
CCG Pharmacist/s
CCG Pharmacy Technician/s
I don't know
Other

4.2.d - Which organisation/s were/are responsible for these activities

AHSN
CCG
PRIMIS
The practice/s
I don't know
Other

4.3.b - Which professional role/s (if known) were/are responsible for these activities?

General practitioner/s
Practice Nurse/s/Advance Nurse Practitioner/s
Practice Manager
Administrator/s
Data lead/s
PCN Pharmacist/s
Practice-based Pharmacist/s
PCN Pharmacy Technician/s
Practice-based Pharmacy Technician
Dispenser/s (in dispensing practices)
CCG Pharmacist/s
CCG Pharmacy Technician/s
I don't know
Other

4.3.d - Which organisation/s were/are responsible for these activities

AHSN
CCG
PRIMIS
The practice/s
I don't know
Other

4.4.b - Which professional role/s (if known) were/are responsible for these activities?

General practitioner/s
Practice Nurse/s/Advance Nurse Practitioner/s
Practice Manager
Administrator/s
Data lead/s
PCN Pharmacist/s
Practice-based Pharmacist/s
PCN Pharmacy Technician/s
Practice-based Pharmacy Technician
Dispenser/s (in dispensing practices)
CCG Pharmacist/s
CCG Pharmacy Technician/s
I don't know
Other

4.4.d - Which organisation/s were/are responsible for these activities

AHSN
CCG
PRIMIS
The practice/s
I don't know
Other

4.5.b - Which professional role/s (if known) were/are responsible for these activities?

General practitioner/s
Practice Nurse/s/Advance Nurse Practitioner/s
Practice Manager
Administrator/s
Data lead/s
PCN Pharmacist/s
Practice-based Pharmacist/s
PCN Pharmacy Technician/s
Practice-based Pharmacy Technician
Dispenser/s (in dispensing practices)
CCG Pharmacist/s
CCG Pharmacy Technician/s
I don't know

Other

4.5.d - Which organisation/s were/are responsible for these activities

AHSN
CCG
PRIMIS
The practice/s
I don't know
Other

4.6.b - Which professional role/s (if known) were/are responsible for these activities?

General practitioner/s
Practice Nurse/s/Advance Nurse Practitioner/s
Practice Manager
Administrator/s
Data lead/s
PCN Pharmacist/s
Practice-based Pharmacist/s
PCN Pharmacy Technician/s
Practice-based Pharmacy Technician
Dispenser/s (in dispensing practices)
CCG Pharmacist/s
CCG Pharmacy Technician/s
I don't know
Other

4.6.d - Which organisation/s were/are responsible for these activities

AHSN
CCG
PRIMIS
The practice/s
I don't know
Other

13.1.a - What is your (and any other individual/s who has been involved in completing the questionnaire) job role?

Practice Nurse/Advance Nurse Practitioner
Practice Manager
Administrator
Data Lead
PCN Pharmacist
Practice-based Pharmacist
PCN Pharmacy Technician
Practice-based Pharmacy Technician
Dispenser
CCG Pharmacist
CCG Pharmacy Technician
Other

13.1.c - How many years have you worked for this practice/s?

less than one year
1-2 years
3-5 years
6-10 years
11-15 years
more than 15 years

13.2.a - What is your (and any other individual/s who has been involved in completing the questionnaire) job role?

Practice Nurse/Advance Nurse Practitioner
Practice Manager
Administrator
Data Lead

PCN Pharmacist
Practice-based Pharmacist
PCN Pharmacy Technician
Practice-based Pharmacy Technician
Dispenser
CCG Pharmacist
CCG Pharmacy Technician
Other

13.2.c - How many years have you worked for this practice/s?

less than one year
1-2 years
3-5 years
6-10 years
11-15 years
more than 15 years

13.3.a - What is your (and any other individual/s who has been involved in completing the questionnaire) job role?

Practice Nurse/Advance Nurse Practitioner
Practice Manager
Administrator
Data Lead
PCN Pharmacist
Practice-based Pharmacist
PCN Pharmacy Technician
Practice-based Pharmacy Technician
Dispenser
CCG Pharmacist
CCG Pharmacy Technician
Other

13.3.c - How many years have you worked for this practice/s?

less than one year
1-2 years
3-5 years
6-10 years
11-15 years
more than 15 years

13.4.a - What is your (and any other individual/s who has been involved in completing the questionnaire) job role?

Practice Nurse/Advance Nurse Practitioner
Practice Manager
Administrator
Data Lead
PCN Pharmacist
Practice-based Pharmacist
PCN Pharmacy Technician
Practice-based Pharmacy Technician
Dispenser
CCG Pharmacist
CCG Pharmacy Technician
Other

13.4.c - How many years have you worked for this practice/s?

less than one year
1-2 years
3-5 years
6-10 years
11-15 years
more than 15 years

13.5.a - What is your (and any other individual/s who has been involved in completing the questionnaire) job role?

Practice Nurse/Advance Nurse Practitioner
Practice Manager
Administrator
Data Lead
PCN Pharmacist
Practice-based Pharmacist
PCN Pharmacy Technician
Practice-based Pharmacy Technician
Dispenser
CCG Pharmacist
CCG Pharmacy Technician
Other

13.5.c - How many years have you worked for this practice/s?

less than one year
1-2 years
3-5 years
6-10 years
11-15 years
more than 15 years

17.1.c - Please specify which organisation/s was/is involved in leading this activity

AHSN
CCG
PRIMIS
Other

17.2.c - Please specify which organisation/s was/is involved in leading this activity

AHSN
CCG
PRIMIS
Other

17.3.c - Please specify which organisation/s was/is involved in leading this activity

AHSN
CCG
PRIMIS
Other

17.4.c - Please specify which organisation/s was/is involved in leading this activity

AHSN
CCG
PRIMIS
Other

17.5.c - Please specify which organisation/s was/is involved in leading this activity

AHSN
CCG
PRIMIS
Other

17.6.c - Please specify which organisation/s was/is involved in leading this activity

AHSN
CCG
PRIMIS
Other

27.1.a - What is your (and any other individual/s who has been involved in completing the questionnaire) job role?

CCG Pharmacist
CCG Pharmacy Technician

Other

27.1.c - How many years have you worked for this CCG?
less than one year
1-2 years
3-5 years
6-10 years
11-15 years
more than 15 years

27.2.a - What is your (and any other individual/s who has been involved in completing the questionnaire) job role?
CCG Pharmacist
CCG Pharmacy Technician
Other

27.2.c - How many years have you worked for this CCG?
less than one year
1-2 years
3-5 years
6-10 years
11-15 years
more than 15 years

27.3.a - What is your (and any other individual/s who has been involved in completing the questionnaire) job role?
CCG Pharmacist
CCG Pharmacy Technician
Other

27.3.c - How many years have you worked for this CCG?
less than one year
1-2 years
3-5 years
6-10 years
11-15 years
more than 15 years

27.4.a - What is your (and any other individual/s who has been involved in completing the questionnaire) job role?
CCG Pharmacist
CCG Pharmacy Technician
Other

27.4.c - How many years have you worked for this CCG?
less than one year
1-2 years
3-5 years
6-10 years
11-15 years
more than 15 years

27.5.a - What is your (and any other individual/s who has been involved in completing the questionnaire) job role?
CCG Pharmacist
CCG Pharmacy Technician
Other

27.5.c - How many years have you worked for this CCG?
less than one year
1-2 years
3-5 years
6-10 years
11-15 years

more than 15 years
